# Editorial: HIV-1 genetic diversity, volume II

**DOI:** 10.3389/fmicb.2022.1007037

**Published:** 2022-08-22

**Authors:** Kok Keng Tee, Michael M. Thomson, Joris Hemelaar

**Affiliations:** ^1^Department of Medical Microbiology, Faculty of Medicine, University of Malaya, Kuala Lumpur, Malaysia; ^2^HIV Biology and Variability Unit, Centro Nacional de Microbiolog, Instituto de Salud Carlos III, Madrid, Spain; ^3^Infectious Disease Epidemiology Unit, Nuffield Department of Population Health, University of Oxford, Oxford, United Kingdom

**Keywords:** HIV, subtype, circulating recombinant form (CRF), unique recombinant form (URF), phylogenetic analysis, recombinant

The HIV pandemic continues to be a major global health problem. In 2021, 38.4 million people were living with HIV worldwide. Despite the increasing availability of antiretroviral therapy (ART) worldwide, around 650,000 deaths and 1.5 million new HIV infections occurred in 2021 (UNAIDS, 2022).

A key characteristic of the HIV pandemic is its extraordinary global genetic diversity. After zoonotic transmission of simian immunodeficiency virus from chimpanzees to humans in the beginning of the twentieth century, HIV-1 group M diversified in central Africa in the first half of the century, leading to distinct subtypes, designated by the letters A, B, C, D, F, G, H, J, K, and L (Robertson et al., 2000; Worobey et al., 2008). The second half of the twentieth century was characterized by the global spread of HIV-1 and ongoing diversification (Tebit and Arts, 2011; Hemelaar, 2012; Faria et al., 2014). Genetic divergence between HIV-1 subtypes is around 17–35% at the amino acid level, depending on the subtypes and genome regions considered (Korber et al., 2001).

HIV-1 genetic variability arises due to the error-prone reverse transcriptase enzyme, which leads to high rates of mutation and recombination. A prerequisite for recombination is that an individual is co-infected with two or more different strains of HIV (Vuilleumier and Bonhoeffer, 2015). Recombinants between subtypes are designated as either circulating recombinant forms (CRFs) or unique recombinant forms (URFs) (Robertson et al., 2000). CRFs are defined as recombinant HIV-1 genomes that are identified in three or more epidemiologically unrelated individuals. URFs refer to unique recombinant sequences without evidence of onward transmission. CRFs are consecutively named, in accordance with an internationally defined nomenclature (Robertson et al., 2000). To date, more than 120 distinct CRFs have been described, a number that continues to increase at pace (Hemelaar et al., 2019, 2020b). CRFs can undergo further recombination with other pure subtypes or recombinants, resulting in secondary recombinants, leading to an increasingly complex array of recombinants (Hemelaar, 2012). The proportion of recombinants has been increasing over time, both globally and in most regions, and recombinants now constitute close to a quarter of all HIV-1 infections (Hemelaar et al., 2020a).

Global molecular epidemiological studies have demonstrated that HIV-1 genetic diversity is extremely complex and evolving (Hemelaar et al., 2019, 2020b). The global spread and evolution of HIV-1 has caused differential global distributions of HIV-1 subtypes, CRFs, and URFs, leading to large regional variation in numbers, types, and proportions of HIV-1 genetic variants. The global HIV-1 epidemic is therefore diversifying and recombinants play particularly important roles in Africa, Asia, and South America (Hemelaar et al., 2020a,b). The diverse distribution patterns of HIV variants are determined by complex factors, including social transmission networks, urbanization, transportation networks, migration, founder effects, and population growth. It may also be that different HIV variants could have an evolutionary advantage in terms of transmission and pathogenesis (Arien et al., 2007; Tebit and Arts, 2011; Faria et al., 2014). Increasing global HIV-1 genetic diversity clearly forms a major obstacle to development of a globally effective HIV-1 vaccine (Gaschen et al., 2002). It also impacts the design of diagnostic, resistance, and viral load assays. Finally, the variability and rapid evolution of HIV-1 provide the means to examine the evolutionary relationships and origins of strains (phylogenetics), the growth dynamics of transmission networks (phylodynamics), and to track the geographical spread of HIV-1 (phylogeography) (Hemelaar, 2012, 2013).

This Research Topic brings together studies that further expand our knowledge of the origins and spread of HIV-1 genetic variants, and examines the impact of HIV-1 diversity on prevention and treatment efforts, including HIV-1 vaccine development and drug resistance.

## Recombination

In this issue, Bacqué et al. conducted a HIV-1 molecular epidemiological study among patients recruited in Spain. A novel CRF derived from subtypes B and F1, designated CRF66_BF, was characterized using whole genome sequencing and detailed phylogenetic and recombination analyses. Bayesian coalescent analyses, which estimated the divergence time of the most recent common ancestors of the sampled genomes, showed that the probable origin of CRF66_BF was in Paraguay around 1984.

Inter-continental transmission of HIV-1 among countries with close socioeconomic relationship is efficient in driving the dissemination of novel recombinants. For example, CRF47_BF of South American origin has expanded considerably in Spain since it was first reported in 2010, with a predominant transmission via heterosexual contact. Hill et al. in this issue revealed that CRF47_BF originated in Brazil, before it spread into Spain and expanded rapidly until the mid-2010s, with evidence of spillover into the men who have sex with men (MSM) population. This and other studies established the repeated introduction and expansion of CRFs in Spain, which highlights the need to establish molecular epidemiological surveillance systems that could provide timely information on cross-border introduction and dynamics of HIV-1 strains.

However, precision in CRF characterization can be compromised by the extreme genome plasticity of HIV-1, in addition to the lack of complete genome sequences and a standardized parameter in recombination analyses. Here, Cañada-García et al. identified a novel HIV-1 BF1 recombinant (CRF122_BF1) in Europe and South America that was previously unidentified (or, rather, misclassified as CRF72_BF1). Apart from different recombination signals detected in the polymerase and envelope genes, CRF122_BF1 was otherwise highly similar to CRF72_BF1 that was described in Brazil. The study highlighted the continuous emergence of new CRFs as a result of co-circulation of multiple viral lineages.

The expanding complexity of HIV-1 recombinants in Africa and Asia are also illustrated in this issue. Among acutely and recently infected patients in Kigali, Rwanda, Umviligihozo et al. reported an increasing frequency of URFs from 23% in a preceding cohort in 2005–2011 to 57% in 2016–2019 that comprised of inter-subtype A1/C and A1/C/D recombinants. Similarly, He et al. reported significant prevalence of URFs among the newly diagnosed MSM population in Shenyang city of Liaoning province, northeast China, between 2016 and 2020, which involved the CRF01_AE/CRF07_BC and CRF01_AE/B recombinants. Taken together, these studies highlight the power of molecular epidemiological surveillance in tracking the evolutionary dynamics of HIV-1 worldwide.

## Molecular epidemiology and phylodynamics

While the study of defined HIV-1 subtypes and recombinants is clearly important, the emergence and expansion of diverse lineages *within* subtypes and CRFs have also become the focus of attention. Such lineages represent viruses sharing a common ancestry propagating within a transmission network, some of which are associated with peculiar biological features (Cid-Silva et al., 2018; Song et al., 2019; Ge et al., 2021; Wymant et al., 2022). The study of the emergence, spatiotemporal propagation, and growth dynamics of HIV-1 variants, based on the analysis of viral sequence evolution, is the subject of phylodynamics. Tracking the expansion of HIV-1 lineages through phylodynamic and phylogenetic methods can inform the design of public health interventions aimed at epidemic control (Brenner et al., 2013; Paraskevis et al., 2016; German et al., 2017; Oster et al., 2018; Vasylyeva et al., 2020).

In this Research Topic several papers fall within the field of HIV-1 molecular epidemiology. Three of them are focused on the use of phylodynamics to track HIV-1 epidemic spread of intra-subtype lineages. Arantes et al. and Arimide et al. focus on estimation of growth rates of lineages circulating in the Amazonas state of Brazil and Ethiopia, respectively. Arantes et al. found continuous expansion until most recent times and comparable epidemic growth rates of Amazonian subtype B non-pandemic [derived from the original subtype B radiation from Haiti (Gilbert et al., 2007)] and pandemic (derived from the subtype B expansion from the USA that disseminated worldwide) lineages. Arimide et al. found sharp declines in transmission parameters in all Ethiopian subtype C lineages coinciding with public health awareness campaigns and behavioral interventions in the mid-1990s, a decade before ART roll-out. On the other hand, Nduva et al. focused on phylogeographic analyses to estimate geographic dissemination of HIV-1 lineages among MSM in Kenya, finding significant dissemination from the Coast to Nairobi and Nyanza provinces and from Nairobi to Nyanza. The public health implications of these results are emphasized by the authors.

The Eastern European and Central Asian region has the fastest growing HIV-1 epidemic in the world, but is insufficiently studied. Sivay et al. examined HIV-1 genetic diversity in Kyrgyzstan, a country in this region for which there were few prior data, analyzing 555 samples. In contrast to most countries in the region, where A6 sub-subtype predominates, in Kyrgyzstan, a Central Asian CRF02_AG variant is predominant, although A6 is also common. No phylogenetic structure was seen in A6, but four geographically-associated lineages were found in CRF02_AG.

The importance of dense sampling for cluster detection and the usefulness of phylogeny for estimating the place of HIV-1 acquisition in migrants is highlighted by Gil et al. who analyzed two densely-sampled Spanish regions, finding an association of clusters with MSM and native Spaniards, but 35% of Latin American immigrants belonged to Spanish clusters (and, therefore, probably acquired HIV-1 in Spain), compared to 1.2% of Sub-Saharan Africans.

While most HIV-1 molecular epidemiology studies focus on the coding regions of HIV, the study by Bhange et al. highlights intra-subtype genetic variation present in the long terminal repeat (LTR) promoter region. In their study of 764 ART-naïve individuals in India they find nine different promoter variant strains of subtype C, which contain additional copies of existing transcription factor binding sites, created by duplication, which may impact viral gene expression and latency.

Methodological improvements for HIV-1 cluster detection are needed in molecular epidemiological studies, and, in line with this, Guang et al. describe a new method based on next generation sequencing incorporating within-host diversity that can detect clusters not detected by consensus sequence approaches.

## Antiretroviral drug resistance

The relationship of antiretroviral (ARV) drug resistance to phylogeny and phylodynamics derives from the fact that HIV-1 drug resistant strains may persist for many years, propagating in phylogenetically-identifiable transmission networks, and from the frequent use of polymerase sequences obtained for drug resistance testing for molecular epidemiology studies. Surveillance of ARV drug resistance transmission is important to monitor the expansion of drug resistant strains, which may affect the choice of first-line ART regimens.

Two papers in this issue focus on such surveillance. Pingarilho et al. analyzed transmitted drug resistance (TDR) and transmission clusters in newly-diagnosed patients in Portugal, finding higher proportions of TDR and clustered TDR among heterosexuals than among MSM, attributing this difference to higher pre-exposure prophylaxis usage and HIV testing among MSM. Miranda et al., using the EuResist database, examined trends of TDR and acquired drug resistance (ADR) in Europe in 1981–2019, comparing late presenters (LP) and non-late presenters (NLP), finding a decreasing trend in both TDR and ADR, and similar TDR frequencies and mutation profiles in LP and NLP.

Monitoring sequence changes in proteins targeted by ARV drugs can inform drug usage in a geographic area and serve as guide to optimize therapeutic choices. Along this line, Bimela et al., using sequences from the Los Alamos HIV Sequence Database, analyzed changes in frequencies of drug resistance mutations (DRM) and naturally occurring polymorphisms in Cameroon, where HIV-1 is highly diverse, before and after implementation of combination ART, finding much more frequent changes in reverse transcriptase than in protease and integrase, mirroring the usage of drugs targeting these enzymes in Cameroon.

The Los Alamos database was also used by Troyano-Hernáez et al. to perform extensive analyses of capsid and polymerase sequences of all circulating HIV-1 genetic forms (groups, subtypes, and CRFs) for variant-specific markers and DRM, finding that mutations in the capsid associated with resistance to lenacapavir (the most promising drug targeting this protein) and major DRM in polymerase in drug-naïve individuals were infrequent in all genetic forms. Valadés-Alcaraz et al. derived HIV transmembrane glycoproteins consensus sequences for subtypes and CRFs and assessed their level of conservation in the different gp41 structural domains, with no natural major resistance mutation to fusion inhibitor T-20 observed.

## Global evolution and vaccines

As highlighted by many studies in this Research Topic, HIV-1 continues to evolve around the world. To reflect this change, Linchangco et al. updated the global whole genome consensus sequences for HIV-1 subtypes and CRFs from 2002 to 2021, based on sequences deposited in the Los Alamos database. Finally, global HIV-1 diversity forms a major obstacle to the development of a globally effective HIV-1 vaccine. Given the genetic divergence between HIV-1 subtypes, it may be necessary to employ subtype-specific vaccines in individual countries according to their HIV-1 subtype distribution. A study by Elangovan et al. estimated the global and regional need for subtype-specific therapeutic and prophylactic HIV-1 vaccines, indicating that to achieve global coverage, HIV-1 vaccines should be mainly directed against subtypes A, B, and C.

## Conclusions

The papers in this Research Topic highlight the incessantly increasing genetic diversification of HIV-1, through the generation of recombinant forms and the emergence and expansion of new lineages. These studies also exemplify the important roles of analyzing the growth dynamics and tracking the geographic spread of HIV-1 variants, through phylogenetic, phylodynamic, and phylogeographic methods, for epidemiological and public health purposes. Another area of interest in this issue relates to the importance of surveying the constantly evolving picture of HIV-1 genetic diversity to inform vaccine immunogen design and to ensure the effectiveness of ARV drugs.

To date, many molecular epidemiology studies have been unsystematic and uncoordinated. There is a need for a coordinated global molecular epidemiological surveillance system that could provide up-to-date, accurate, and geographically representative information on the evolution and spread of HIV variants to aid prevention and treatment efforts. At present, most subtyping is done as an adjunct to resistance testing, performed by *pol* sequencing. This means that samples are often unrepresentative of populations and recombinants may be missed, as no information is available outside *pol*. Representative sampling from key populations and/or the general population will be essential and will depend on the state of the HIV epidemic in each country (Aldrich and Hemelaar, 2012). Moreover, whole genome sequencing is crucial to adequately characterize HIV strains and detect recombinants. With the increasing availability of deep sequencing, whole genome sequencing is becoming increasingly feasible, and also enhances detecting dual/multiple infections and minority drug-resistant strains (Aldrich and Hemelaar, 2012).

The use of phylogenetic and phylodynamic methods in public health is increasingly being advocated, and, in fact, the plan for ending the HIV epidemic in the USA includes the use of such tools for rapid HIV-1 outbreak detection and response (Fauci et al., 2019). However, further empirical data on the effectiveness of such strategies are needed. More research is needed on the impact of HIV-1 diversification and the increasing proportion of recombinants on transmission and pathogenesis. Finally, in order to end the global HIV epidemic, it is imperative that the recent successes in developing COVID vaccines are harnessed to accelerate HIV-1 vaccine development, with immunogen sequence design that accounts for global HIV-1 diversity.

## Author contributions

All authors contributed equally to the writing of this editorial and read and approved the final version of the manuscript.

## Conflict of interest

The authors declare that the research was conducted in the absence of any commercial or financial relationships that could be construed as a potential conflict of interest.

## Publisher's note

All claims expressed in this article are solely those of the authors and do not necessarily represent those of their affiliated organizations, or those of the publisher, the editors and the reviewers. Any product that may be evaluated in this article, or claim that may be made by its manufacturer, is not guaranteed or endorsed by the publisher.
